# Multiple Participants’ Discrete Activity Recognition in a Well-Controlled Environment Using Universal Software Radio Peripheral Wireless Sensing

**DOI:** 10.3390/s22030809

**Published:** 2022-01-21

**Authors:** Umer Saeed, Syed Yaseen Shah, Syed Aziz Shah, Haipeng Liu, Abdullah Alhumaidi Alotaibi, Turke Althobaiti, Naeem Ramzan, Sana Ullah Jan, Jawad Ahmad, Qammer H. Abbasi

**Affiliations:** 1 Research Centre for Intelligent Healthcare, Coventry University, Coventry CV1 5FB, UK; syed.shah@coventry.ac.uk (S.A.S.); ad4828@coventry.ac.uk (H.L.); 2 School of Computing, Engineering and Built Environment, Glasgow Caledonian University, Glasgow G4 0BA, UK; syedyaseen.shah@gcu.ac.uk; 3 Department of Science and Technology, College of Ranyah, Taif University, P.O. Box 11099, Taif 21944, Saudi Arabia; a.alhumaidi@tu.edu.sa; 4 Faculty of Science, Northern Border University, Arar 91431, Saudi Arabia; turke.althobaiti@nbu.edu.sa; 5 School of Computing, Engineering and Physical Sciences, University of the West of Scotland, Paisely PA1 2BE, UK; naeem.ramzan@uws.ac.uk; 6 School of Computing, Edinburgh Napier University, Edinburgh EH10 5DT, UK; s.jan@napier.ac.uk (S.U.J.); j.ahmad@napier.ac.uk (J.A.); 7 James Watt School of Engineering, University of Glasgow, Glasgow G12 8QQ, UK; qammer.abbasi@glasgow.ac.uk

**Keywords:** USRP, RF sensing, software-defined radio, multi-subject monitoring, smart healthcare, ensemble learning

## Abstract

Wireless sensing is the utmost cutting-edge way of monitoring different health-related activities and, concurrently, preserving most of the privacy of individuals. To meet future needs, multi-subject activity monitoring is in demand, whether it is for smart care centres or homes. In this paper, a smart monitoring system for different human activities is proposed based on radio-frequency sensing integrated with ensemble machine learning models. The ensemble technique can recognise a wide range of activity based on alterations in the wireless signal’s Channel State Information (CSI). The proposed system operates at 3.75 GHz, and up to four subjects participated in the experimental study in order to acquire data on sixteen distinct daily living activities: sitting, standing, and walking. The proposed methodology merges subject count and performed activities, resulting in occupancy count and activity performed being recognised at the same time. To capture alterations owing to concurrent multi-subject motions, the CSI amplitudes collected from 51 subcarriers of the wireless signals were processed and merged. To distinguish multi-subject activity, a machine learning model based on an ensemble learning technique was designed and trained using the acquired CSI data. For maximum activity classes, the proposed approach attained a high average accuracy of up to 98%. The presented system has the ability to fulfil prospective health activity monitoring demands and is a viable solution towards well-being tracking.

## 1. Introduction

Due to a wide variety of applications, human activity sensing (detection or monitoring) has received immense attention in recent years. The goal is to identify how humans react and behave especially in enclosed environments. Some of the applications where human activity sensing is highly considered are: smart home systems, context-aware systems, Internet-of-Things (IoT) systems, and healthcare systems, in particular for the monitoring of elderly people [1,2]. Lately, non-invasive human activity sensing schemes have been proposed based on Software-Defined Radio (SDR) [3], WiFi [4], and Radio Detection and Ranging (RADAR) technology [5]. The key characteristic of the human activity sensing system is the human body, which is primarily water (up to 60%) and capable of reflecting radio signals. As a result, the adjacent radio system’s received signal properties change and indicate distinct human activities.

In most conventional human activity detection systems, either wearable devices or camera-based technologies are employed [6,7]. Although these technologies are highly accurate, nevertheless, certain limitations are associated with them. For instance, camera-based technology possesses the risk of privacy, and wearable devices may cause discomfort while being attached to a body for long periods. Therefore, a non-invasive (or contactless) technology is required such as Radio-Frequency (RF) sensing, which has limited privacy concerns and does not require being attached to a body. Over the years, research on RF sensing technology has provided several advantages over conventional methods [8]. For instance, RF-based methods do not require the placement of any sensors on the human body. The reflection of the wireless signals from the human body is used to assess different actions of humans. As a result, patients such as pregnant women, children, and the elderly will find it easier to be monitored by contactless technology [9,10].

The majority of current research on human activity sensing has focused on single subjects, whereas research on multiple subjects is still in its early stages [11,12]. With the advancement of microelectronics and IoT technologies, sensors are increasingly becoming prevalent in everyday life. According to estimates from the Information Handling Services company, the IoT industry will increase from 15 billion devices deployed in 2015 to 30 billion in 2020 and about 75 billion in 2025 [13]. A vast range of information can be obtained with the help of these smart devices, which can be useful in a variety of fields, including public safety, businesses, and healthcare.

In this paper, we focused on multi-subject activity sensing using Universal Software Radio Peripheral (USRP) devices. The USRP devices are based on SDR technology employed for distinct RF applications. To the best of our knowledge, this research work is the first of its kind to utilise non-invasive technology for multi-subject activity sensing considering a realistic environment. In general, multi-subject activities can be classified into three categories as per the tasks that subjects perform [14]:

- **Group activity:** In a group activity, two or more subjects perform a common task simultaneously, for example two subjects performing a walking activity;
- **Multi-individual activity:** In a multi-individual activity, two or more subjects perform tasks that are not related. For example, one subject performs the sitting activity, while another subject performs the standing activity;
- **Mixed activity:** In a mixed activity, both above-mentioned activities are considered, for example two subjects at the same time performing the walking activity, while one subject performs the sitting activity.

The rest of this paper is organised in the following manner: Section 2 provides up-to-date information about wireless sensing technologies. Section 3 presents the proposed scheme based on SDR sensing. Section 4 presents experimental outcomes, and lastly, Section 5 provides concluding remarks and recommendations for further study.

## 2. Related Work

This section compares existing wireless sensing techniques, which were effectively utilised in the past for various applications, including human activity sensing. These technologies are based on the Channel State Information (CSI) approach, the Received Signal Strength Indicator (RSSI) approach, the RADAR approach, and the SDR approach.

The CSI-based approach exploiting WiFi technology has recently become prevalent for feature extraction in human-related activity recognition [15,16,17]. Several research works have focused their efforts on creating CSI-based applications such as human presence detection [18], human crowd reckoning [19], indoor localisation [20], and a fall/collapse detection scheme for the elderly and young [21]. According to some of the current literature, WiFi signals are capable of detecting and distinguishing even tiny movements of the human body, for instance mouth motions [22], keystrokes [23], heart rate [24], and respiratory rate [25].

The RSSI-based approach for human activity sensing is primarily dependent on the received signal strength variations induced by distinct human actions [26]. In comparison to SDR and CSI, the RSSI-based system has limited detecting ability and precision. The SDR-based method improves the identification accuracy by up to 72%, allowing for a better resolution of RSSI capture [27]. With the lack of frequency diversity, which is present in CSI-based systems, the precision and coverage region of RSSI-based systems are lower. The RSSI is recorded by a single value per packet, while the CSI is assessed per Orthogonal Frequency-Division Multiplexing (OFDM) from each packet. This makes the CSI approach more stable and provides further information compared to RSSI. As a result, CSI is more robust towards challenging situations.

The RADAR-based approach with a significantly greater bandwidth is also employed for human activity recognition [28,29]. In contrast to WiFi-based technology, Frequency-Modulated Continuous Wave (FMCW) RADAR exploits a bandwidth up to 1.79 GHz, while WiFi technology only uses a bandwidth up to 20 MHz [30]. The RADAR-based methods are used for micro-Doppler information extraction and have a greater distance resolution of around 20 cm [31,32]. Nevertheless, RADAR-based solutions need specialised processing units and hardware.

The SDR-based approach is a specifically built hardware that can be employed to sense several human activities [33,34]. WiSee utilises USRP to detect the Doppler shifts in wireless signals and accomplish activity identification with an accuracy of up to 94% [35]. Using specialised circuit hardware, Allsee technologies introduced a short-range detection technique for gesture recognition that is less than 2.5 ft [36,37]. The sole method for extracting wireless CSI from WiFi signals without upgrading or modifying the hardware is to employ a platform based on SDR [38].

## 3. Methodology

**Figure 1:**
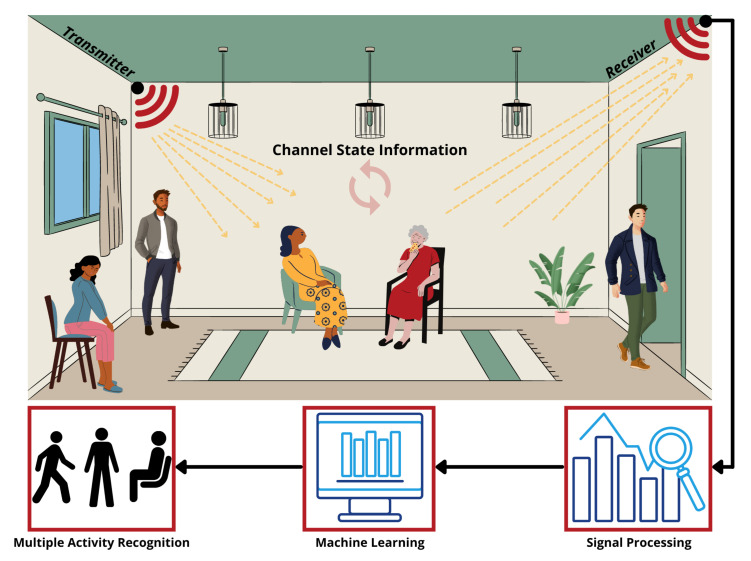
Proposed system concept design based on non-contact sensing technology and AI for multiple individuals’ activity recognition.

This section presents a complete methodology adopted to carry out this research. It begins with a paragraph on the experimental scheme and proceeds on to a thorough overview of the hardware design stage, data acquisition, data wrangling, and proposed system model training based on the Machine Learning (ML) ensemble approach. Figure 1 depicts the proposed system’s overall concept and primary components. The conceptual design, as depicted, is based on a non-invasive sensing system that can detect the presence and activity of several individuals in the same space. Moreover, integrating a wireless sensing system with advanced Artificial Intelligence (AI) algorithms can assist in identifying a variety of human activities in real time.

### 3.1. Experimental Scheme

**Figure 2:**
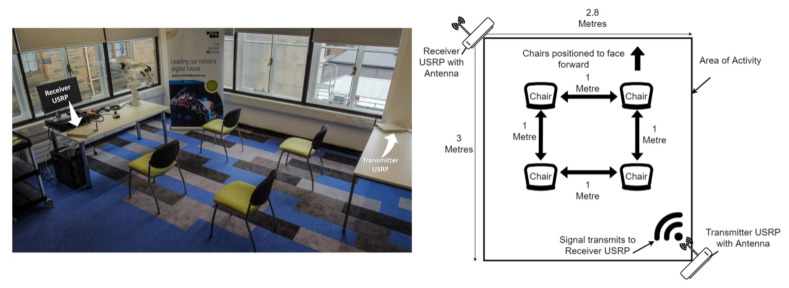
Setup for recording distinct activities utilising the 5G frequency in an experimental setting [39].

To confirm the efficacy of the proposed methodology, a number of experiments were conducted in a rectangular activity area of 2.8 × 3 m${}^{2}$, as shown in Figure 2. In the opposite corners, two X-Series USRP devices for CSI signal transmission and receiving were placed. During multiple experiments, the subjects (or participants) changed positions arbitrarily in the specified activity area while maintaining a one-meter distance between themselves to capture maximal intraclass variance for all activities, including sitting, standing, and walking. This scenario was performed to mimic a diminutive setting as in an elderly care facility with a small group of individuals. The experiment’s current focus was on accommodating four individuals; however, this number will be increased in the future research work. Furthermore, the proposed ML-based classification approach to recognise multiple participants’ activities is made up of two primary modules: model training and model testing. The model training module uses an offline approach to train the ML algorithm using previously obtained and preprocessed CSI samples data. The model testing took place in an online environment, where an input CSI data sample was categorised as one of the human activities after all essential preprocessing was completed. The two modules are described in detail in Section 4.

**Figure 3:**
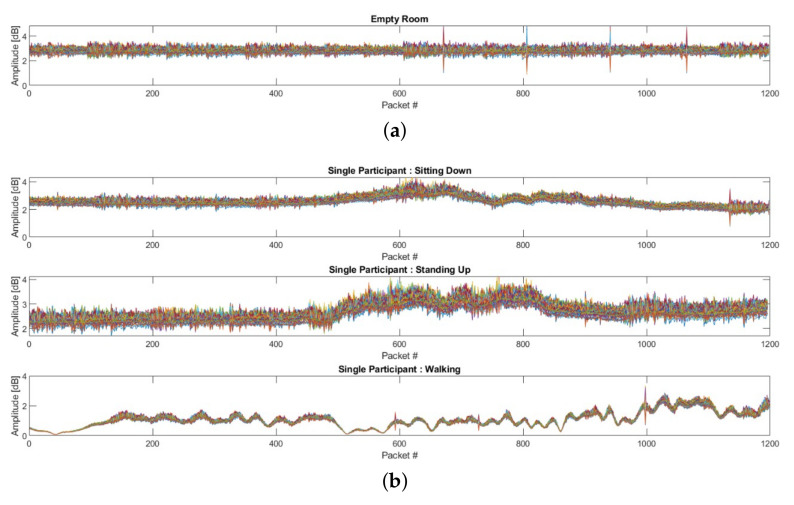

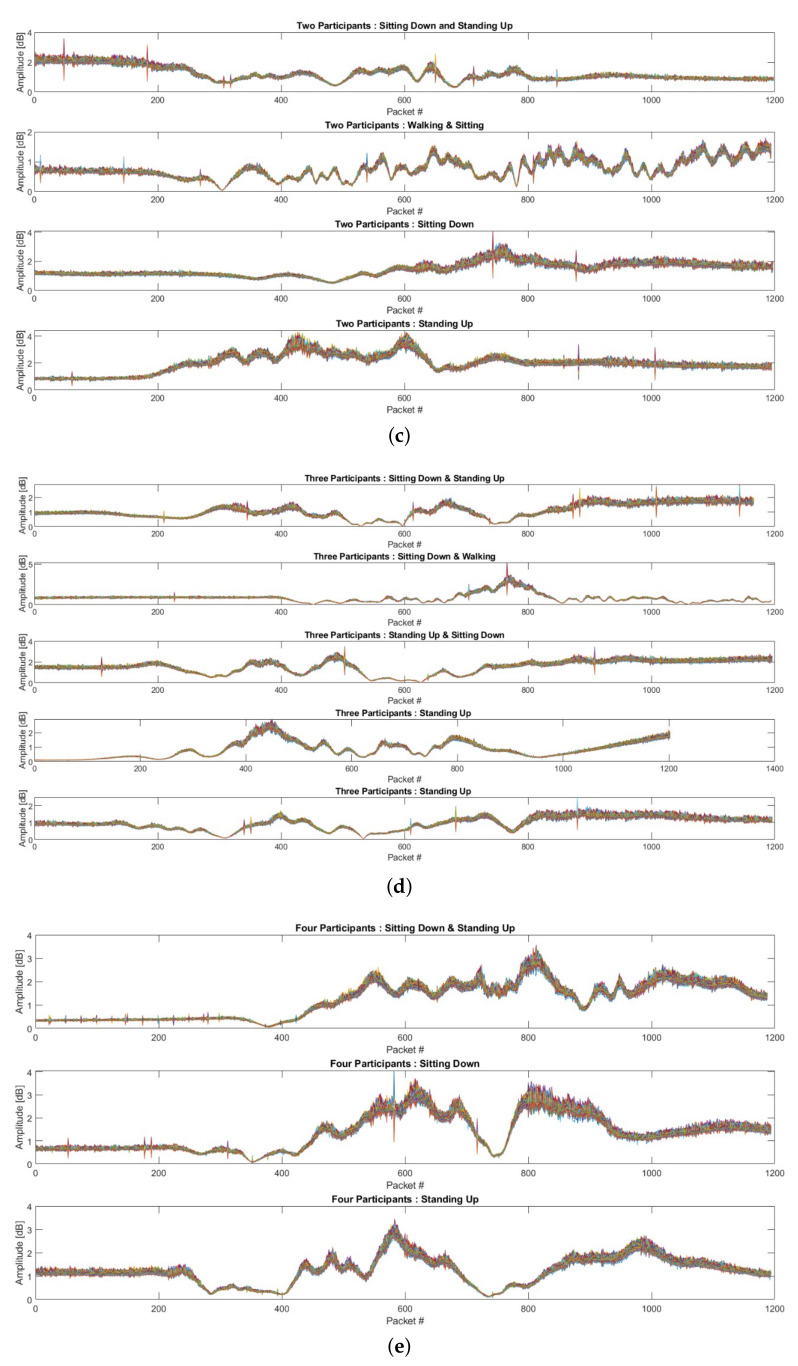
Samples of obtained CSI data from different activities: (**a**) no participant (empty room), (**b**) 1 participant, (**c**) 2 participants, (**d**) 3 participants, and (**e**) 4 participants (see Table 1).

In one of the previous works [39], a similar CSI dataset was adopted, and experiments were carried out in multiple phases to identify different human activities. The information regarding the dataset is provided in Table 1 (see Figure 3 for the data samples). Each data sample of the activity consists of approximately 50×1200 data points. For instance, 100 data samples are equivalent to 100×50×1200 data points. To recognise multi-subject activities, a deep-learning-based solution called the “convolutional neural network” was designed and trained in the afore-mentioned paper. The classification accuracy and confusion matrix were used as evaluation metrics. The employed approach attained an average accuracy of 91.25% for single-subject activity and an overall 83% accuracy for four subjects’ activities segregated into sixteen distinct classes. In order to improve the performance of recognising multiple activities based on CSI data, we performed certain enhancements, which are described as follows.

1. Instead of deep learning, we adopted an ensemble-learning-based solution in this paper. The ML ensemble technique is explained in Section 3.5, while Section 4 reveals the experimental outcomes based on an ensemble approach for the recognition of multiple activity classes;
2. In order to design a lightweight scheme that can be effectively utilised for the real-time system, we exceedingly reduced the data points from the original dataset. The details regarding the employed data for training and testing are provided in the first paragraph of Section 4;
3. Instead of assessing a classifier’s efficiency based on accuracy and a confusion matrix, we exploited six distinct criteria to evaluate the performance of the trained ML classifiers, including accuracy, precision, recall, F1-score, confusion matrix, and model training duration. The reason for selecting multiple evaluation criteria is that classifiers can fail to correctly identify some classes, even if the overall accuracy is higher (e.g., due to excessive false positives and lower true positives). However, the precision will decrease significantly.

### 3.2. Hardware Design

The experiment carried out in this research study made use of two USRP devices, each outfitted with the VERT2450 omnidirectional antenna. One USRP served as the transmitter, while the other served as the receiver. Each USRP was linked to a separate PC with 16 GB RAM and an Intel Core i7-3.60 GHz CPU. To provide the Ubuntu 16.04 operating system, the system made use of a Virtual Machine (VM). GNU Radio was employed to interact with the USRPs on the Ubuntu VM. The USRP function can be carried out using flow diagrams generated using GNU Radio. Following that, the flow diagrams can be transformed to Python scripts. A single Python script was used to send data to the transmitter, while another received data from it. The transmitter used OFDM to send random integers from 0–255. The transmitter sent a signal to the receiver, which was configured to receive it. The CSI complex number was then output to the terminal by the script, which ran on the receiver side. Following that, the amplitude readings from the CSI complex numbers were taken from this output. Table 2 lists the system’s primary configuration parameters.

### 3.3. Data Acquisition

The acquisition of data for the proposed scheme’s model training comprised five phases, with four participants performing three distinct activities (sitting, standing, walking) in a laboratory setting, as illustrated in Figure 2. The setup was duplicated in two distinct laboratory settings to introduce varying amounts of complexity, increasing the volatility of the data and strengthening the system model. Nonetheless, the data for a specific activity class gathered in both laboratory settings were handled as a single dataset, indicating that the amount of complexity caused by the surroundings was not a measurable variable in the experiments carried out.

The same as any other experiment, there were both fixed and variable characteristics in this experimental study. The fixed characteristics for the experiment presented in this study were: the hardware and its setup; the data wrangling and ML approaches; the experimental setting. The variable characteristics were: the number of participants; the participant identity; the position of the performed activity (e.g., one participant performing the sitting activity in various chair positions, as shown in Figure 2). The first variable was assessed, and the outcomes are emphasised in the rest of this section and in the findings, whereas the second and third variables were used to establish the utmost intraclass variance in the obtained data. To ensure data reproducibility across different days, all data were collected over the course of a week, with a randomised amount of data taken for each of the 16 classes on each day of the week. The activities *sitting* and *standing* depict the action of conducting these tasks rather than the posture/position of an individual in the sitting or standing condition. The participants were not compelled to maintain their upper body motionless while recording the activity data; therefore, both the *sitting* and *standing* activity data contained minor shifts of the upper body. The details regarding each activity employed in this paper are listed in Table 1 and explained in the following paragraph.

In the first data acquisition stage, a single participant’s CSI data were gathered separately for the sitting, standing, and walking activities, where 420 samples were obtained in total. Three distinct participants took part in the data acquisition phase to ensure maximal variance. Each participant contributed evenly to the data acquisition, i.e., each participant took part in the acquisition of one-hundred forty samples, which were split among the three activities classes. In 3 s, 1200 packets were transmitted for each CSI data sample. In the second data acquisition stage, two participants performed the three activities indicated above, for a total of 400 samples. This data acquisition step featured the same three individuals with equal contributions, i.e., from each participant, a minimum of thirty-three samples were acquired per the four classes chosen for this phase of data acquisition. Three and four volunteers were enrolled in the third and fourth data acquisition stages to assist in the collecting of data on multiple activities simultaneously. The individuals recruited for this data acquisition phase remained the same throughout. In this phase, 540 and 300 data samples were obtained in total. Additionally, 117 samples of data were acquired for the class “Empty”, which depicts the state of the space when the participants are not present. Each of the 16 classes are listed in Table 1. Figure 3 shows the data samples from the distinct activities. The interclass variance in the data samples from various activity classes was evident, and it can be used in the classification procedure to improve the outcomes.

### 3.4. Data Wrangling

As stated in Equation (1), the CSI for a single transmitter and a receiver antenna produces a matrix, including frequency reactions for all N=51 subcarriers.

(1) $$H={\left[H_{1}\left(f\right),H_{2}\left(f\right),\ldots ,H_{N}\left(f\right)\right]}^{T}$$

The frequency here per subcarrier $H_{i}$ can be written as:

(2) $$H_{i}\left(f\right)=\left|H_{i}\left(f\right)\right|e^{j∠H_{i}\left(f\right)}$$

The *i*th subcarrier amplitude and phase responses are represented by $\left|H_{i}\left(f\right)\right|$ and $∠H_{i}\left(f\right)$. Each of these subcarrier responses relates to the system input and output, as provided in Equation (3).

(3) $$H_{i}\left(f\right)=\frac{X_{i}\left(f\right)}{Y_{i}\left(f\right)}$$

where $X_{i}\left(f\right)$ and $Y_{i}\left(f\right)$ are the Fourier transforms of the system’s input and output, respectively.

The obtained CSI data were generally obscured as a result of high-frequency ambient noise and multi-path CSI signal propagation. As a result, the data were sent through the subsequent data processing or wrangling phases in order to denoise them and prepare them for the ML training process:

1. In the initial phase, as shown in Equation (4), each sample of CSI data was averaged over all 51 subcarriers to obtain a single averaged data sample for consequent processing.
  (4) $$x_{i}=\frac{1}{J}\sum_{j=1}^{J}y_{ij}$$
  where $x_{i}$ is the *i*th data sample, which portrays an average across equivalent subcarriers $y_{ij}$ for (j=1,2,3,…,51);
2. After that, each averaged data sample $x_{i}$ was smoothed and minor variations were removed using the Butterworth lowpass filter of order n=4;
3. Subsequently, the approximation coefficients $A_{i}$ for every smooth data sample $s_{i}$ were obtained utilising the discrete wavelet transform with a Haar basis function at Level 3. Since the approximation coefficients reflect the turnout of the lowpass filter in the discrete wavelet transform, it further assisted the noise reduction. The downsampling and convolution processes entailed in the wavelet decomposition across all three extents are expressed mathematically as follows:
  (5) $$A_{i}^{0}\left[m\right]=\sum_{k=0}^{M-1}s_{i}\left[k\right]\times g\left[2m-k\right],\;\mathrm{for}\;m=1,2,\ldots ,M$$
  (6) $$A_{i}^{1}\left[t\right]=\sum_{k=0}^{T-1}A_{i}^{0}\left[k\right]\times g\left[2t-k\right],\;\mathrm{for}\;t=1,2,\ldots ,T=\frac{M}{2}$$
  (7) $$A_{i}^{2}\left[u\right]=\sum_{k=0}^{U-1}A_{i}^{1}\left[k\right]\times g\left[2u-k\right],\;\mathrm{for}\;u=1,2,\ldots ,U=\frac{M}{4}$$
  where g[k] for k=1,2,3,…,K represents the lowpass filter of length *K* per decomposition level, $s_{i}\left[m\right]$ for m=1,2,3,…,M represents the smooth signal of length *M* after implementing the Butterworth lowpass filter, and $A_{i}^{l}$ for levels l=0,1,2 represents the approximation coefficient of three levels of the discrete wavelet transform.

### 3.5. Ensemble-Technique-Based Training

The purpose of the ML-based ensemble technique is to aggregate the predictions of several base estimators created using a specific learning algorithm to increase generalisation and robustness over a single estimator. Averaging is one of the methods used in the ensemble technique, where the idea is to build numerous estimators independently and then average their estimates. Since its variance is decreased by this approach, the merged estimator is generally enhanced compared to any of the single base estimators [40].

**Figure 4:**
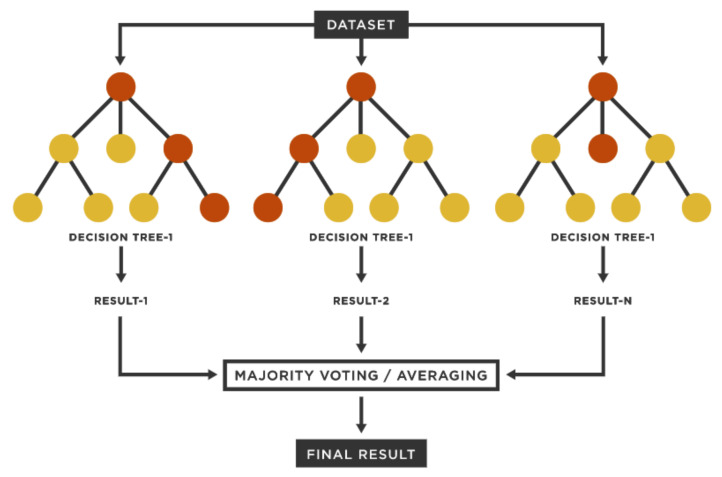
Ensemble-technique-based classification approach.

The Extremely Randomised Trees or Extra Trees (ETs) and the Random Forest (RFo) algorithm are two averaging approaches based on randomised Decision Trees (DTs). Both approaches are perturb and merge methods created particularly for trees. This shows that by inserting randomness into the ML classifier building, a varied group of classifiers is created. The averaged prediction of the individual classifiers was used to obtain the ensemble prediction. Figure 4 illustrates the ensemble method, which consisted of multiple DTs. An individual tree is composed of a root node, child nodes, and leaf nodes.

In RFo, each tree in the ensemble is generated using a sample selected with replacement from the training data. The optimal split is selected from all input features or a random subset of size max features when dividing each node during tree building. On the other hand, randomness is taken a step further in the case of ETs. A random subset of features is utilised in the same way as RFo; however, rather than looking for the most discriminating thresholds, thresholds are produced at random for each feature. The best of these randomly produced thresholds is chosen as the splitting criteria. This generally allows for a small reduction in model variance at the risk of a slight increase in bias. In this paper, we used both of the ensemble techniques (ETs/RFo) for classification purposes and compared their performance with DTs.

## 4. Experimental Findings

All simulations for this work were performed on a MacBook Air with a processor 1.6 GHz Dual-Core Intel Core i5 and memory 8 GB 1600 MHz DDR3. The Python programming language was utilised for the ML part, primarily using the scikit-learn, NumPy, and pandas libraries. As described earlier in this paper, the data were acquired based on 16 different activity classes (see Table 1). Out of the total acquired data for each activity, we exploited 3600×1200 data points or observations. Considering all 16 activity classes (16×3600), a total of 57600×1200 data points were used for experimental purposes. We considerably reduced the data from the original datasets in order to design a lightweight solution that can be efficiently used for real-time schemes. From the total data points, 70% were used for training the ML models and 30% for testing. The hyperparameters for training the ML algorithms were obtained through the grid-search technique. These hyperparameters are revealed in Table 3.

Using a single performance-examining criterion for the algorithms is typically not a good practise in the ML world. If the classifier somehow does not accurately identify some classes, the accuracy may still be greater, for instance high false positives and low true positives. Nevertheless, the precision will decline significantly. As a result, evaluating a classifier’s effectiveness only based on accuracy is inadequate. Therefore, to examine the performance of the trained ML classifiers in this work, we used six different criteria, such as accuracy, precision, recall, F1-score, confusion matrix, and training time of the models.

**Figure 5:**
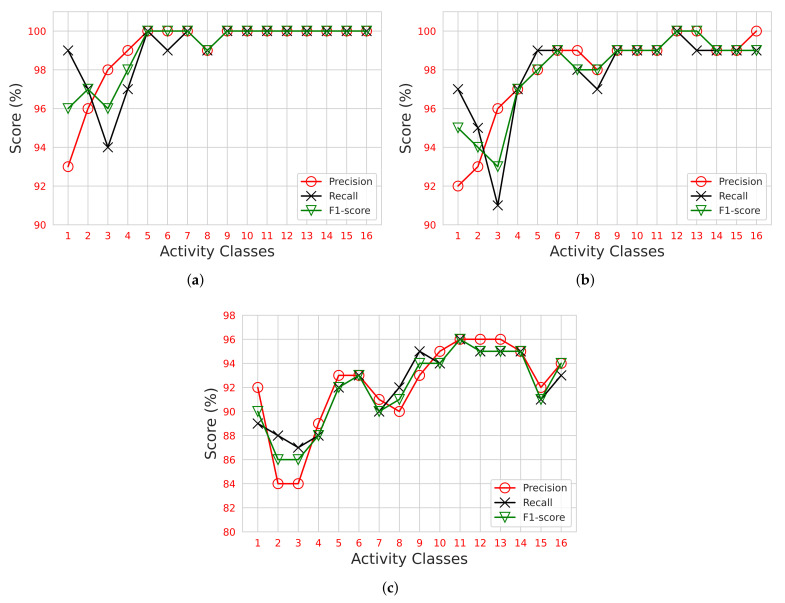
Precision, recall, and F1-score comparison for individual activity classes on: (**a**) extra tree; (**b**) random forest; (**c**) decision tree.

In Figure 5a, the ET performance can be noted for the precision, recall, and F1-score. For all 16 activity classes, the ET scored more than 92%. From Classes 9–16, the ET attained up to a 100% score for all evaluation metrics. The minimum score noted was 93% precision for the first class, that is an empty room. In Figure 5b, the performance of RFo can be noted. Although many variations can be seen for all 16 activity classes, nevertheless, RFo scored more than 90% for each distinct class. The minimum score noted was 91% recall for the third class, that is one person standing. The maximum score noted with all three evaluation metrics was 100% for the twelfth and thirteenth classes, which were three persons sitting and three persons standing. In Figure 5c, the DT performance can be noted. The minimum (second and third class) and maximum (eleventh, twelfth, and thirteenth class) scores attained by the DT were 84% and 96%, respectively. This shows how the DT can perform classification tasks well; nevertheless, an ensemble of DTs (ET/RFo) can perform even better by the combination of multiple DTs.

**Figure 6:**
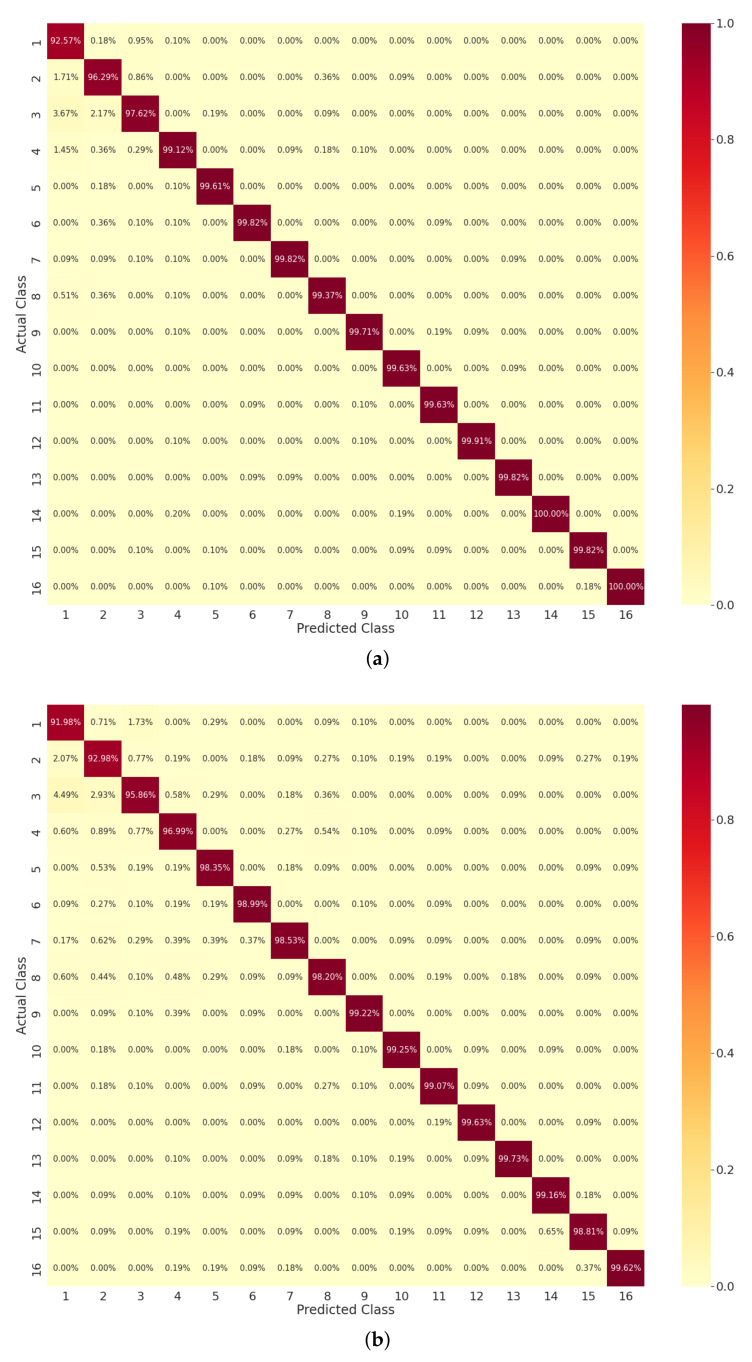

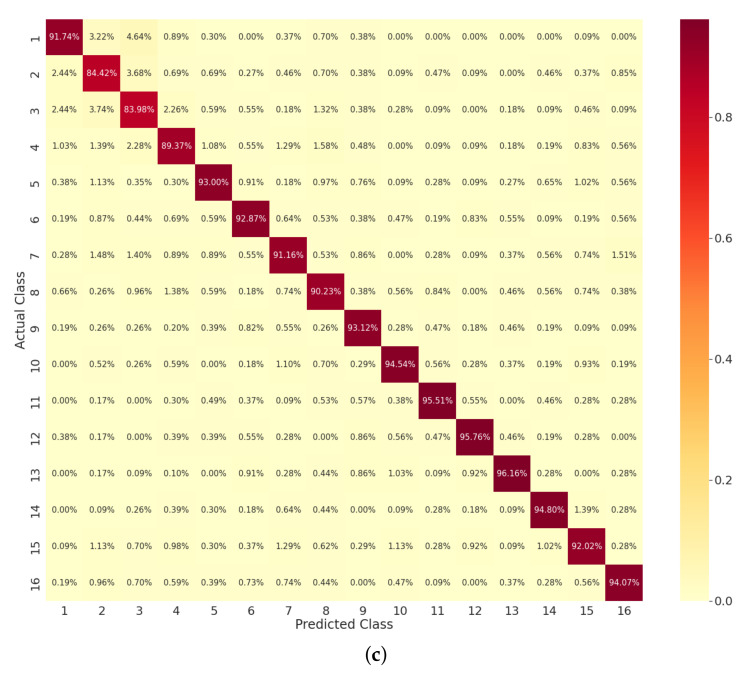
Confusion matrix report of sixteen distinct activity classes by trained model: (**a**) extra tree; (**b**) random forest; (**c**) decision tree.

In Figure 6a, the ET performance can be seen in the form of a confusion matrix. From Classes 4–16, the ET accomplished a maximum prediction score of about 99%. Some of the classes such as the first, second, and third had a few misclassifications that resulted in a prediction score of 92%, 96%, and 97%, respectively. In Figure 6b, the performance of RFo can be observed. As shown, RFo accomplished up to 99% correct prediction scores for the activity classes: ninth (1 sitting + 2 standing), tenth (1 walking + 2 sitting), eleventh (2 sitting + 1 standing), twelfth (3 sitting), thirteenth (3 standing), fourteenth (4 sitting), and sixteenth (2 sitting + 2 standing). The class with the maximum misclassification and minimum prediction score was the first one (empty room). The possible reason for that is the data features of the first class and how it was different from rest of the classes, which were entirely based on some activity happening. In Figure 6c, the DT performance can be noted. For most of the activity classes, the DT obtained more than a 90% prediction score except a few activity classes: second (1 sitting), third (1 standing), and fourth (1 walking). As can be noted, the DT held more misclassifications compared to the ET and RFo.

Furthermore, Table 4 presents the overall classification accuracy of ML classifiers for all 16 activity classes and the average training time of the models in terms of seconds. We exploited the cross-validation method using the parameter cv=5 to check the overall accuracy of the algorithms for all activity classes. The cross-validation approach randomly splits a set of data observations into *k* groups, or folds of roughly comparable size. The technique was applied to the remaining k−1 folds, with the first fold functioning as a validation set. Cross-validation is an approach utilised in applied ML to evaluate the model’s competence on unknown data. It is a well-known strategy since it produces a less biased assessment of the ML model compared to other approaches.

As shown in Table 4, the ET accomplished the highest accuracy up to 98% with a minimal training time of 2.24 s. RFo attained up to 97% accuracy with a training time of about 10 s. The DT reached the utmost accuracy rate of 90% with the highest training time of around 120 s. These results reveal how an ensemble-based ML approach can be effectively used for classification tasks. The randomisation technique in ensemble approaches (i.e., ET) improves the tree’s diversity and makes it easier to reduce the correlation when developing the trees. Ensemble learning approaches can be described as divide-and-conquer techniques or the wisdom of the crowd. Stable and more robust ML models can be obtained with accurate predictions utilising an ensemble method since it highly reduces the bias and variance errors. As a result, the ET approach can be emphasised for classification and regression tasks. The quick training time of the ET demonstrates how it can be utilised for a vast number of applications, especially real-time lightweight systems for hospitals and care centres.

## 5. Conclusions and Future Work

The objective of this study was to propose a contactless RF sensing system for detecting and monitoring human presence and simultaneously activities (such as sitting, standing, and walking) utilising CSI signals. The system operated at 3.75 GHz, that is within the 5G frequency range of 3.4–3.8 GHz. The primary aim was to propose a non-invasive sensing system that could identify the presence and activity of multiple humans in the same space. The findings of this paper reveal that integrating RF sensing technology with state-of-the-art ensemble-based machine learning algorithms (such as extra trees) can efficiently recognise diverse human activities of daily living. The system was put to the test to see how well it could recognise simultaneous activities among a variety of subjects, ranging from 0–4. Variations were added to train the system on 16 various activities, simulating a realistic real-life scenario. The activity recognition experiments revealed that all test participants’ activities were recognised with up to 98% accuracy. The findings of this study are intriguing and have much potential for real-world applications.

The goal for future research is to expand the experiments to cover the majority of the human activity spectrum in different spaces with the maximum number of subjects. Experiments will be carried out to see how the number of spaces affects the performance of the proposed scheme. Additionally, various heights and placements of the transmitter/receiver antennas will be investigated.

## Figures and Tables

**Table 1:** Number of participants and performed activities (see Figure 3 for the data samples).

No. of Participants	No. of Classes	Activities Performed	No. of Data Samples
0	1	Empty Room	117
1	2	1 Sitting	140
	3	1 Standing	140
	4	1 Walking	140
2	5	1 Sitting + 1 Standing	100
	6	1 Walking + 1 Sitting	100
	7	2 Sitting	100
	8	2 Standing	100
3	9	1 Sitting + 2 Standing	120
	10	1 Walking + 2 Sitting	120
	11	2 Sitting + 1 Standing	100
	12	3 Sitting	100
	13	3 Standing	100
4	14	4 Sitting	100
	15	4 Standing	100
	16	2 Sitting + 2 Standing	100

**Table 2:** Parameter selection and configuration of the software.

Parameter	Value
Platform	USRP X300/310
OFDM Subcarriers	51
Operating Frequency	3.75 GHz
Transmitter Gain	70 dB
Receiver Gain	50 dB

**Table 3:** Machine learning algorithms’ hyperparameters adopted by the grid-search method for training.

Classifier	Hyperparameters
Extra Tree	bootstrap = False ccp − alpha = 0 class − weight = None criterion = gini max − depth = None max − features = auto max − leaf − nodes = None max − samples = None min − impurity − decrease = 0 min − impurity − split = None min − samples − leaf = 1 min − samples − split = 2 min − weight − fraction − leaf = 0 n − estimators = 5 n − jobs = None oob − score = False random − state = None verbose = 0 warm − start = False
Random Forest	bootstrap = True ccp − alpha = 0 class − weight = None criterion = gini max − depth = None max − features = auto max − leaf − nodes = None max − samples = None min − impurity − decrease = 0 min − impurity − split = None min − samples − leaf = 1 min − samples − split = 2 min − weight − fraction − leaf = 0 n − estimators = 5 n − jobs = None oob − score = False random − state = None verbose = 0 warm − start = False
Decision Tree	ccp − alpha = 0 class − weight = None criterion = gini max − depth = None max − features = None max − leaf − nodes = None min − impurity − decrease = 0 min − impurity − split = None min − samples − leaf = 1 min − samples − split = 2 min − weight − fraction − leaf = 0 presort = deprecated random − state = None splitter = best

**Table 4:** Classifiers’ average training time and overall accuracy score by cross-validation using the parameter cv=5.

Classifier	Training Time	Accuracy
Extra Tree	2.24 s	98%
Random Forest	10.52 s	97%
Decision Tree	120.61 s	90%

## Data Availability

Not applicable.
